# Advances on the Role of Lung Macrophages in the Pathogenesis of Chronic Obstructive Pulmonary Disease in the Era of Single-Cell Genomics

**DOI:** 10.7150/ijms.100160

**Published:** 2025-01-01

**Authors:** Xiaohua Li, Hui Zhang, Xianhong Chi, Weibin Ruan, Xia Meng, Jiehua Deng, Mianluan Pan, Tingting Ma, Jianquan Zhang

**Affiliations:** 1 Department of Respiratory and Critical Medicine, the Eighth Affiliated Hospital, Sun Yat-Sen University, Shenzhen 518000, Guangdong Province, China.; 2 Department of Respiratory and Critical Medicine, The First Affiliated Hospital of Guangxi Medical University, Nanning, Guangxi 530021, China.; 3 Department of Respiratory and Critical Medicine, Zhuhai People's Hospital (Zhuhai Hospital affiliated with Jinan University), Zhuhai, Guangdong 519000, China.

**Keywords:** macrophages, chronic obstructive pulmonary disease, immune function, scRNAseq, cigarette smoke

## Abstract

Chronic Obstructive Pulmonary Disease (COPD) is a heterogeneous respiratory disorder characterized by persistent airflow limitation. The diverse pathogenic mechanisms underlying COPD progression remain incompletely understood. Macrophages, serving as the most representative immune cells in the respiratory tract, constitute the first line of innate immune defense and maintain pulmonary immunological homeostasis. Recent advances have provided deeper insights into the phenotypic and functional alterations of pulmonary macrophages and their role in COPD pathogenesis. Notably, the advent of single-cell RNA sequencing has revolutionized our understanding of macrophage molecular heterogeneity in COPD. Herein, we review principal investigations concerning the sophisticated mechanisms through which pulmonary macrophages influence COPD, encompassing inflammatory mediator production, protease/antiprotease release, and phagocytic activity. Additionally, we synthesize findings from available literature regarding all identified pulmonary macrophage sub-populations in COPD, thereby advancing our comprehension of macrophage heterogeneity's significance in the complex pathophysiological mechanisms of COPD.

## Introduction

Chronic Obstructive Pulmonary Disease (COPD) represents a complex respiratory disorder characterized by persistent airflow limitation and respiratory manifestations. These clinical features stem from two primary pathological changes: airway structural abnormalities (bronchitis and bronchiolitis) and parenchymal destruction (emphysema) 1. The global burden of COPD continues to escalate, with particularly concerning trends in low- and middle-income countries. This increasing prevalence stems from demographic shifts toward an aging population, combined with inadequate regulation of tobacco use and environmental pollutants, resulting in substantial socioeconomic impact 2,3. COPD has become the third leading cause of death worldwide 4, emphasizing the critical need to elucidate its pathogenic mechanisms and identify novel therapeutic targets.

The pathological processes underlying COPD development encompass several interconnected mechanisms, including inflammatory response 5,6, dysregulation of protease-antiprotease balance 7, and tissue destruction 8. Current evidence demonstrates remarkable tissue-specific heterogeneity among macrophages, as exemplified by microglia, osteoclasts, and Kupffer cells. Nevertheless, the extensive overlap in surface marker expression has impeded the precise identification and classification of different macrophage populations 9.

The emergence of single-cell RNA sequencing (scRNAseq) has transformed our understanding of macrophage diversity. This advanced methodology employs next-generation sequencing platforms to conduct comprehensive transcriptome analysis at single-cell resolution, enabling detailed examination of cellular gene expression patterns. In comparison to conventional bulk RNA sequencing approaches, scRNAseq offers superior resolution and accuracy in characterizing individual cell transcriptional profiles, facilitating the identification of both prevalent and rare cellular populations 10. The application of scRNAseq has expanded across multiple disease contexts, providing valuable insights into macrophage diversity and immunological changes in endocrine disorders 11, hematological malignancies 12, neurological conditions 13, gastrointestinal diseases 14,15,16, and cardiovascular pathologies 17. However, a comprehensive review of scRNAseq applications in COPD pathogenesis is lacking.

This review presents a three-dimensional analysis of macrophage involvement in COPD pathogenesis and summarizes the underlying regulatory mechanisms to identify potential therapeutic interventions. Furthermore, we provide a detailed characterization of macrophage sub-populations identified through scRNAseq, illustrating their roles in various mechanisms of COPD and underscoring the importance of macrophage heterogeneity in COPD initiation and progression.

## Macrophage function and COPD

**Figure 1:**
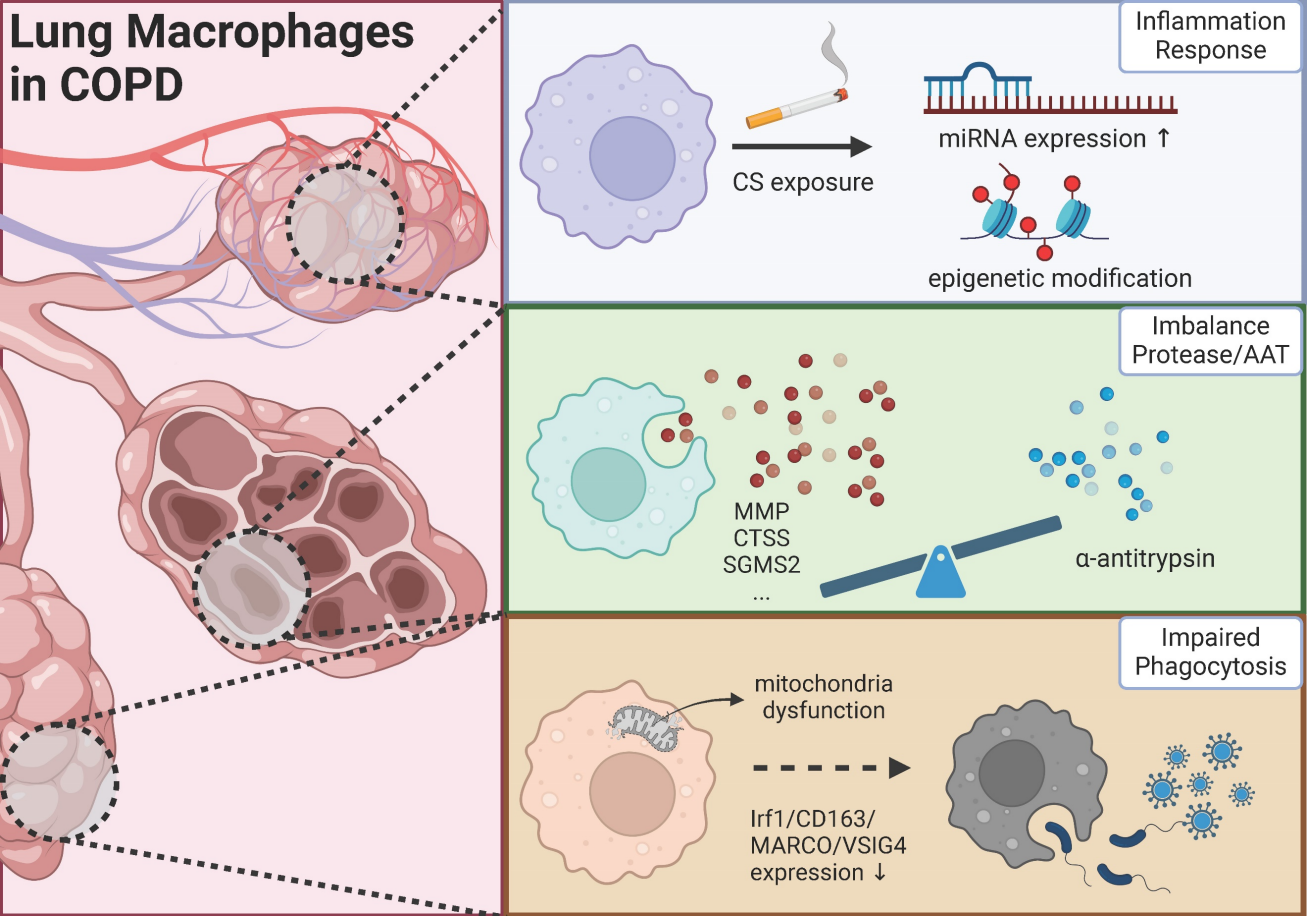
The role of lung macrophage in the progression of COPD (Created in BioRender: *https://BioRender.com/b40o000*).

This review endeavors to elucidate the complex pathogenic mechanisms of COPD from three distinct perspectives: chronic inflammation, protease/antiprotease balance, and phagocytic activity, with particular emphasis on macrophage involvement in these processes. As depicted in ***Figure 1***, macrophages assume diverse roles across different mechanisms, and macrophages exhibiting similar functions may represent distinct cellular sub-populations.

### Gene expression regulation in macrophages: a critical component of COPD inflammatory mechanisms

For one thing, as a key immune cell type, macrophages play a broad role in the pathophysiology of chronic inflammatory diseases. Following chemokine signals, macrophages migrate to inflammatory sites and undergo polarization through complex signaling networks, thus coordinating inflammatory responses. For another, chronic inflammation remains a major focus in understanding COPD mechanisms. Current treatments for COPD are mainly limited to symptom management, such as airway dilation, highlighting the pressing need for new therapeutic approaches. With recent advances in biological therapies, many studies have focused on inflammatory cells and factors as potential therapeutic targets for COPD, and several clinical trials of COPD-specific biological agents are ongoing. However, the complete picture of how macrophages regulate inflammation in COPD remains unclear. We review recent studies from a new perspective of gene regulation, examining how macrophages control COPD inflammation through miRNA and epigenetic modifications.

MicroRNAs (miRNAs) are small, non-coding RNA molecules, typically comprising approximately 23 nucleotides in length, that lack the capacity for translation into proteins 18. Extensive research has demonstrated that miRNA are widely involved in cellular biological processes, thereby maintaining tissue and organ homeostasis. Conversely, dysregulation of miRNA expression can lead to disease onset and progression. Recent studies have begun to uncover the specific mechanisms by which miRNA in pulmonary macrophages regulate chronic lung inflammation, particularly in COPD. Specifically, *miR-21-3P* exhibits heightened expression levels in alveolar macrophages (AMs) of mice exposed to cigarette smoke (CS) 19. Moreover, elevated levels of miR-21 and miR-155 are observed in lung samples from both smokers with and without COPD, as well as in macrophages exposed to cigarette smoke extract (CSE) 20,21. Significantly, epithelial cell exocytosis is found to decrease the secretion of miR-21 in response to higher concentrations of CSE 22, which suggests a potential role for *miR-21* in regulating apoptosis and inflammation, thereby implicating its involvement in the pathogenesis of CS-induced COPD. Notably, variations in miRNA expression profiles among lung macrophages across distinct lung regions, attributed to variances in oxygen levels, indicate the necessity of integrating spatial miRNA levels for a comprehensive evaluation of macrophage functionality 23. Investigating the mechanistic involvement of miRNA in macrophages in COPD may serve as a valuable approach for targeted diagnostic and therapeutic strategies, leveraging the inherent stability of miRNAs and the advanced technology of miRNA high-throughput sequencing to enhance the precision and sensitivity of miRNA detection. Furthermore, the analysis of miRNA-mRNA interactions may offer insights into cellular interactions within the context of COPD. For instance, a study employs next-generation sequencing and bioinformatics analyses to identify the miR6511a-5p-NT5E interaction 24, highlighting the intimate relationship between bronchial epithelial cells and macrophages in the airway micro-environment of COPD. Based on these collective findings, we propose that investigating the mechanistic involvement of miRNAs in COPD macrophages can be enhanced by leveraging both the inherent stability of miRNA and advanced high-throughput sequencing technologies, which together improve the precision and sensitivity of miRNA detection. Furthermore, miRNA-containing extracellular vesicles are emerging as promising novel biomarkers for early COPD diagnosis and severity assessment 25. However, current studies provide limited evidence regarding the regulatory role of macrophage-derived, miRNA-carrying extracellular vesicles in COPD inflammatory mechanisms.

Furthermore, epigenetic modifications also play a significant role in the pathogenesis of COPD. Specifically, N6-methyladenosine (m6A) is an epigenetic modification that regulates gene expression and function by impacting post-transcriptional RNA modifications 26. The increased gene of m6A modifications in lung tissues of both stable COPD and acute exacerbation of COPD (AECOPD) mice indicates that differentially methylated genes are implicated in signaling pathways related to immune function 26. Methylation in macrophage genes in individuals with COPD plays a significant role in the development of the disease 1,27. Analysis of genome-wide methylation patterns in sputum cells indicates that methylation of genes in macrophages in COPD primarily affects those associated with Major Histocompatibility Complex (MHC) class I and class II molecules linked to human leukocyte antigen (HLA) genes 28. These findings underscore the potential impact of methylation on the inflammatory response within the lungs. Methylation is intricately linked to monocyte recruitment in COPD, as indicated by epigenetic networks that propose the involvement of the macrophage epigenetic factor protein arginine methyltransferase 7 (PRMT7) in the transcription and monomethylation of the RAP1A-regulated histone protein, which is crucial for monocyte recruitment 29,30. Furthermore, in conjunction with methylation alterations, acetylation modifications play a significant role in the development of COPD. Through the examination of transcriptomic, proteomic, and acetylomic data obtained from mice model of COPD induced by CS, it is observed that aldolase A (ALDOA) is notably downregulated and hyperacetylated in the lung tissues of both COPD patients and mice 31, which suggests that ALDOA may serve as a promising diagnostic tool and/or biomarker for COPD. The decreased expression of key enzymes involved in the novo NAD^+^ synthesis pathway in macrophages, including HAAO, KMO, KYNU, and QPRT, in the chronic particulate matter (PM)-induced mouse model of COPD results in reduced expression of the kynurenine pathway and subsequent histone acetylation, and it implied that histone acetylation is responsible for the increased expression of proinflammatory genes in macrophages exposed to PM 32. Targeting metabolic pathways in macrophages and manipulating epigenetic reprogramming represent potential novel strategies for treating COPD 32. While epigenetic editing is anticipated to induce enduring changes in gene expression, further investigation is required to comprehensively elucidate the impact on the intrinsic chromatin landscape.

### Macrophage-associated proteases/protease inhibitors and COPD

In 1997, Hautamaki *et al.* 33 first demonstrated in animal models that pulmonary macrophages release elastase, a key factor in CS-induced emphysema. This discovery prompted an investigation into the types and production mechanisms of these elastases. Subsequently, Finlay *et al.* 34 found that AMs in bronchoalveolar lavage fluid (BALF) samples from emphysema patients secreted high levels of proteases, including matrix metalloproteinases (MMPs), leading to elastolysis and damage of alveolar tissue.

With technological advances, the diversity of these proteases and their associated mechanisms have been progressively revealed. Currently, MMPs, primarily synthesized by macrophages, are widely recognized as the principal proteases in emphysema, particularly MMP-12 35. Patients with COPD show elevated MMP-12 protein levels in sputum compared to non-COPD individuals 36, and their lungs contain a higher number of MMP-12-positive macrophages 37. Mechanistically, MMP-12 enhances placental growth factor (PGF) expression and upregulates its downstream signaling molecules, resulting in bronchial epithelial cell apoptosis and emphysema development 38. Targeting the activity of transient receptor potential mucolipin 3 (TRPML3) affects endolysosomal trafficking and phagocytosis of MMP-12 in pulmonary macrophages 39, ultimately reducing COPD-related emphysema incidence. Beyond MMP-12, other MMP family members including MMP-9 and MMP-1 have been documented in the literature 40,41. Notably, some researchers have proposed that macrophages with high MMP-12 expression represent distinct cellular sub-populations in atherosclerosis 42. We suggest that AMs might similarly be classified into sub-populations, although COPD research has yet to establish clear definitions for such classifications.

Other proteases merit significant attention. Cathepsin S (CTSS), an elastolytic protease, shows increased expression in macrophages during COPD progression, leading to exacerbated lung tissue damage 43. Additionally, proteinases with a disintegrin and a metalloproteinase domain (ADAMs) are intricately linked with macrophages. Specifically, in smokers and even more so in COPD patients, lung macrophages exhibit heightened and detrimental expression of ADAM9 44, often resulting in tissue damage and promoting emphysema development. Notably, ADAM8 45, an enzyme known for its protective properties, is found to be reduced in individuals with COPD, Additionally, ADAM8 deficiency has been shown to exacerbate lung inflammation by inhibiting CS-induced activation of the intrinsic apoptosis pathway in macrophages, although its expression in macrophages is still undetermined.

Conversely, protease inhibitors play a vital role in maintaining protease balance. Alpha-1 antitrypsin (AAT), encoded by the *SERPINA1* gene, functions as an elastase inhibitor that modulates pulmonary inflammatory responses. A large-scale, multicenter retrospective study demonstrated significant survival benefits of plasma-purified AAT intravenous therapy in patients with severe AAT deficiency (AATD) 46. However, research indicates that individuals with AATD, particularly those with genetic mutations resulting in insufficient AAT levels, exhibit elevated expression of matriptase (a membrane-bound serine protease) in their AMs 47. This elevation leads to extracellular matrix degradation and pulmonary tissue destruction. Additionally, matriptase may regulate MMP-14 activity, indirectly contributing to lung extracellular matrix degradation. These findings not only partially explain the limitations of AAT supplementation therapy but also identify potential therapeutic targets.

### Impaired phagocytosis of macrophages in COPD

Lung macrophages play a dual role in maintaining pulmonary homeostasis by facilitating the clearance of apoptotic cells to prevent the release of intracellular toxic substances and promoting tissue repair, while also participating in the phagocytosis of particles and microorganisms to orchestrate the immune response during infection.

In individuals with COPD, the phagocytosis of lung macrophage is often compromised 48. Proteomic analysis of lung tissues has revealed a downregulation in the expression of CD163, MARCO, and VSIG4 in macrophages, indicating a decrease in their phagocytic activity 49. Recent scRNAseq studies have identified various causes of macrophage phagocytic impairment, including smoking, microenvironment, methylation etc. 50. Specifically, smoking has been shown to disrupt the bronchial-mucus barrier, leading to changes in cellular composition and transcriptome as well as increased mucus production 51. The alteration in the microenvironment results in epigenetic reprogramming of airway macrophages, ultimately affecting the expression of Irf1 and subsequently reducing macrophage autophagy and phagocytosis 52. Reduced methylation has been found to potentially play a role in the defective efferocytosis of AMs in patients with COPD 53. Numerous studies have indicated that mitochondrial dysfunction is a significant factor in the impaired phagocytosis of macrophages in COPD, characterized by proton leakage and reductions in maximal and spare respiratory capacity, coupling efficiency, and the bioenergetic health index, reflecting the overall status of mitochondria 54. The upregulation of Iron-responsive element-binding protein 2 (IRP2) results in increased mitochondrial iron accumulation and cytochrome c oxidase (COX) levels, ultimately leading to mitochondrial dysfunction and the development of experimental COPD 55. The NADPH-generating enzyme malic enzyme 1 (ME1) has been shown to restore mitochondrial function and energy metabolism, effectively reversing phagocytic dysfunction in lung macrophages affected by COPD. ME1 is identified as a target of Nuclear factor erythroid 2-related factor 2 (NRF2), with selective activation of NRF2 resulting in the reduction of reactive oxygen species (ROS), enhancement of energy status, maintenance of redox homeostasis, and restoration of mitochondrial biological characteristics and energy metabolism 56,57. The findings that mice treated with mitochondrial complexing agents or placed on a low-iron diet were shielded from CS-induced COPD indicate a significant involvement of the mitochondrial iron axis in COPD pathogenesis, highlighting its potential as a therapeutic target 55. Furthermore, the expedited degradation and reduced half-life of Rubicon, a crucial protein in phagosomal vesicle formation via the lysosomal pathway, impaired phagocytosis in macrophages 58. The improvement of the *Wnt5a-rac1*-disheveled autophagy pathway linked to phagocytosis in macrophages leads to the restoration and enhancement of macrophage phagocytosis of bacteria 59. However, the use of corticosteroids commonly utilized in the treatment of COPD does not ameliorate lung macrophage phagocytosis dysfunction 60, and the underlying cause of this phenomenon remains uncertain.

The compromised phagocytic function of macrophages in individuals with COPD contributes to the colonization of microbial pathogens in the lower respiratory tract. For instance, patients with COPD exhibit impaired clearance of Haemophilus influenzae by AMs 61, and one factor is the delayed secretion of the antimicrobial peptide S100A8/A9 by epithelial cells, which possesses phagocytosis-enhancing properties 62. Notably, AECOPD is commonly precipitated by respiratory infections, most of which are viral 63. In a retrospective study in Korea, approximately 41.2% of severe acute exacerbations are attributed to viral infections, with human rhinovirus (HRV) being one of the most commonly identified viruses 64. HRV impairs the ability of lung macrophages in patients with COPD to phagocytose bacteria while having no impact on phagocytosis in macrophages of healthy individuals 65. Moreover, the impact of the human immunodeficiency virus (HIV) on phagocytosis in lung macrophages in COPD is evident through the increased presence of poorly phagocytosed double-negative (CD40^-^ CD163^-^) lung macrophages 66. It implies that viral infections exacerbate phagocytosis deficiencies of macrophages in patients with COPD. Notably, derivatives of Tanshinones (TS) have been shown to mitigate the exacerbation of COPD caused by viral infection, with TS demonstrating beneficial effects dependent on hemagglutinin in macrophages 67. However, further research is needed to fully understand the causal relationship between viruses and phagocytosis dysfunction in macrophages in COPD. All in all, the colonization of microbiota in individuals with COPD varies depending on the underlying causes, with an observed correlation between the degree of impairment in phagocytosis and the increased load of respiratory pathogens 68. It highlights the importance of investigating microbial colonization patterns based on the clinical phenotypes of COPD. The heightened cytophagocytosis of black carbon (BC) or carbon content by airway macrophages as potential predictors of respiratory symptoms and impaired lung function following exposure to environmental pollutants like indoor PM2.5 or CS exposure presents a novel avenue for diagnosis of COPD 69,70. Further research is warranted to investigate the intricate relationship and underlying mechanisms between pathogenic microorganisms and the phagocytosis of lung macrophages in COPD. Addressing the dysfunction of phagocytosis in lung macrophages may potentially mitigate the advancement of COPD. Restoring macrophage phagocytosis, in conjunction with innovative strategies such as novel antimicrobials, vaccines, or phage therapeutics to rebalance the airway microbiota, could present a promising avenue for future therapeutic interventions.

## Macrophage diversity in COPD

Although the inflammatory mechanisms of COPD have been increasingly detailed, current therapeutic approaches remain largely limited to symptomatic treatments such as bronchodilators and ventilators, rather than targeting inflammation directly. This limitation partly stems from the marked heterogeneity of pulmonary inflammation in COPD, characterized by variations in inflammatory chemokines, predominant inflammatory cells, and inflammation intensity. These factors create complex inflammatory networks, potentially explaining the differential treatment responses among patients.

### Classical classification of macrophages in COPD

Previous studies have identified four distinct macrophage phenotypes: M0 (non-polarized), M1, M2, and M1-M2 (double-positive) 71-74. M1 macrophages exhibit pro-inflammatory characteristics and are typically associated with inflammatory micro-environments 74, while M2 macrophages are characterized by their anti-inflammatory properties and are predominantly found in normal lung tissue and during inflammation resolution 73,74. For instance, *Mycobacterium tuberculosis* infection intensifies macrophage polarization responses to CS toward both M1 and M2 phenotypes 75. In COPD, significant increases in the proportion of both M1 and M2 macrophages may indicate disease progression 76,77.

Numerous studies have not only confirmed this finding but also revealed potential targets within M2 macrophages. COPD severity can be indirectly assessed by monitoring levels of TREM-2, an immunoregulatory receptor involved in M2 macrophage polarization and phenotype switching, and the TREM-2/TREM-1 ratio 78,79. Peroxisome proliferator-activated receptor γ (PPARγ) agonists enhance M2 macrophage function by inhibiting JAK-STAT, MAPK, and NF-κB pathways 76,80. However, conflicting studies suggest that PPARγ transcription may be impaired in macrophages 80. Increased M2 macrophage proportions lead to enhanced epithelial-mesenchymal transition (EMT), a process closely associated with CS-activated TGF-β/Smad signaling in M2 macrophages 79. Notably, bronchial epithelial cells counteract reduced EMT by decreasing miR-21 content in extracellular vesicles (EVs), thereby reducing M2 macrophage polarization 22.

These findings highlight the limitations of the classical M1/M2 polarization balance theory in explaining macrophage alterations in COPD patients' lungs. Some studies directly suggest that a substantial proportion of macrophages cannot be classified within this dichotomous framework, particularly in healthy individuals and COPD patients 48, 81-82. Significantly, Yu *et al.* 42 synthesized recent single-cell RNA sequencing data from atherosclerosis studies, offering new insights into the traditional M1/M2 classification while proposing additional macrophage sub-populations. This suggests the need to seek new perspectives in understanding the complex macrophage polarization regulatory mechanisms in COPD, with scRNAseq potentially offering a promising approach.

### Macrophage diversity in COPD revisited in the era of single-cell omics

In 2009, Tang *et al.* 83 achieved the first parallel detection of single-cell mRNA through scRNAseq, marking the beginning of the single-cell sequencing era. Subsequently, scRNAseq technology has rapidly evolved and been increasingly applied to cell atlas construction, exploration of cellular heterogeneity and sub-populations, and biomarker discovery. This advancement has expanded our understanding of macrophage heterogeneity: Mulder *et al.* 84 utilized scRNAseq to reveal macrophage diversity in health and disease, suggesting this technology's potential in designing personalized macrophage immunotherapy strategies. Furthermore, scRNAseq has elucidated how various pulmonary cells participate in COPD pathogenesis at single-cell resolution, revealing additional potential therapeutic targets. Lee *et al.* 85 conducted a meta-analysis of scRNAseq datasets, mapping the associations of diverse immune cells in COPD etiology, thereby enhancing our understanding of COPD pathological pathways. Xu *et al.* 86 employed scRNAseq to identify a cathepsin L (CTSL)-secreting eosinophil population crucial in mouse emphysema development, proposing CTSL as a potential therapeutic target. Consequently, we believe that the emergence of scRNAseq technology can address some limitations of classical macrophage classification by identifying additional macrophage sub-populations, ultimately revealing more comprehensive immunoregulatory mechanisms. Here, we present a multi-dimensional analysis of how scRNAseq demonstrates pulmonary macrophage diversity in COPD patients, while also highlighting current limitations in this research direction. Additionally, we have compiled these studies in ***Table 1*** to provide readers with a more intuitive understanding of the rich diversity of macrophage sub-populations.

A study conducted scRNAseq on BALF samples from healthy non-smokers and COPD patients, reveals the presence of four distinct macrophage sub-populations in COPD 81, and the classification of CD206^+^ macrophages based on their autofluorescence levels was achieved by integrating flow cytometry and bulk RNA sequencing (bulk-seq) methodologies, resulting in the identification of CD206^+^ autofluorescenthigh AMs (tissue resident-derived) and CD206^+^ autofluorescentlow AMs (monocyte origin) 81. In a research investigation involving 18 patients diagnosed with COPD and 28 control subjects, the monocytes/macrophages present in the pulmonary tissues of the participants were observed to be classified into 16 distinct sub-populations, among these sub-populations, three primary classes of monocyte/macrophage populations were found to be prevalent in individuals with COPD, including elevated expression levels of FABP4, CD52, and IL-1β respectively, implying a severe inflammatory reaction 82. These findings indicate that scRNAseq offers a superior level of resolution in comparison to conventional methodologies. Furthermore, the diversity of lung macrophages is observed to vary across different stages of COPD. Analysis of scRNAseq from BALF in early COPD revealed the presence of distinct macrophage sub-populations exhibiting elevated expression of genes related to proliferation, histocompatibility II, and hemoglobin following the phagocytosis of erythrocytes 48. This phenomenon has been corroborated in other studies 87.

ScRNAseq is utilized for pseudo-temporal analysis to infer cell trajectories and RNA speeds, providing a deeper examination of the dynamic changes in macrophages during various disease stages of COPD or its different sub-populations, which is advantageous for identifying potential therapeutic targets and compounds. For instance, through a comparison of cell dynamics in healthy and COPD samples, novel therapeutic targets for COPD were discovered in this study, for example, inhaled corticosteroids and other compounds can modulate the transcriptomic profile of distinct lung macrophages and monocytes in patients with COPD 82. But the research contributes valuable insight, because in comparison to research in other disciplines, there is a scarcity of studies on the various stages of COPD development, warranting further investigation. In addition, COPD frequently presents comorbidities with other diseases, typically exhibiting heterogeneity of macrophages. One such comorbidity is Asthma and COPD Overlap Syndrome (ACOS). ScRNAseq reveals that monocytes/macrophages are the predominant cell type in lung tissues of patients with ACOS, constituting over 50% of the cells examined 88.

Numerous academic studies have demonstrated that the integration of various omics techniques has enhanced the efficacy of diagnosing and treating diseases. In the context of COPD, utilizing induced sputum metabolomics/lipidomics can yield a diagnostic framework that effectively discriminates between asthma and COPD 89. By refining and amalgamating protein-protein interaction (PPI), proteomics, and transcriptomics data pertinent to COPD, predictive models for disease classification can be further developed 90, thereby enhancing the precision of analyses conducted on limited sample sizes utilizing multi omics data, and previous studies have also reported such findings 91. Omics approaches—including transcriptomics, epigenetics, whole-exome and wholegenome sequencing—have identified rare genetic determinants of COPD. These findings enhance our understanding of disease susceptibility and the heterogeneity observed among patients 92. The integration of scRNAseq is anticipated to enhance the accuracy of disease diagnosis and treatment 93. In the future, the integration of scRNAseq technology with other omics techniques will be instrumental in elucidating the pathogenesis of macrophages in COPD.

The utilization of scRNAseq in the medical field has experienced notable growth over the last decade. Emerging technologies derived from scRNAseq, such as spatial transcriptomics (ST) platforms like 10^x^ Visium and NanoString GeoMx, offer intricate insights into the spatial heterogeneity of lung macrophage sub-populations 94,95. However, the restricted resolution of these platforms hinders the comprehensive characterization of lung macrophages. It is noteworthy that Stereoseq and DBiT Seq exhibit enhanced resolution and diversity 96-98, thereby offering more comprehensive spatial information regarding mRNA and protein molecules at the individual cell level. Furthermore, ATAC-seq represents a novel methodology utilizing transposase to pinpoint chromosomal regions suitable for transcription 99,100. A study utilizes ATAC-seq to assess the enrichment of COPD risk variants and investigate the regulatory impact of chromatin-level information on lung cells 101. These methodologies offer insights into the spatial and functional heterogeneity of various cell sub-populations.

Henceforth, it is imperative to integrate additional novel technologies in order to investigate the spatial diversity, and molecular and functional diversity of lung macrophages, and gain a more comprehensive understanding of the intricate regulation of lung macrophages sub-populations in COPD.

To sum up, there is a limited number of current studies utilizing scRNAseq to analyze macrophages in COPD, with small sample sizes being a common limitation. A consensus on the classification of macrophages in COPD has yet to be established. Future research should focus on expanding the use of scRNAseq in conjunction with other methodologies to compare macrophage populations from various origins, as well as refining and standardizing criteria for monocyte/macrophage classification. Simultaneously, it is imperative to thoroughly consider the diverse nature of COPD and its associated comorbidities in order to investigate the role of macrophages in the pathogenesis of the disease. This exploration may lead to the identification of potential therapeutic targets for precise intervention and modification of the disease's trajectory, thereby enhancing the progress of personalized therapy.

## Conclusion

This review comprehensively examines the role of pulmonary macrophages in COPD pathogenesis, with particular emphasis on the impact of altered macrophage immune functions, including inflammatory mediator secretion, protease/antiprotease release, and phagocytic activity on COPD progression. Furthermore, we highlight the diversity of pulmonary macrophages in the COPD microenvironment revealed through scRNAseq. The discovery of these novel sub-populations has broadened our understanding of immune regulation in COPD. We propose that leveraging innovative technologies and methodologies to enhance our comprehension of pulmonary macrophage mechanisms in COPD is crucial for developing precision medicine strategies for COPD treatment.

## Figures and Tables

**Table 1:** Overview of macrophage subsets and correspondent marked genes by scRNAseq.

Reference	Sample Source	Macrophage Subsets: Marker Genes (Function)
Liégeois *et al.* 2022 81	BALF in healthy nonsmokers, smokers without COPD, and smokers with COPD	Cluster 1: FAPB4, AKR1C1, LIMA1 (lipid metabolism) Cluster 2: NCF1, ALOX5AP, CES1 (response to toxic substances) Cluster 3:SPP1, SELENOP, MMP9 (matrix components): Cluster 4: MKI67, PCLAF, CDK1 (cell cyclic)
Hu *et al.* 2023 82	Explanted lung tissue of COPD	COPD-predominant: AMs: Cluster 0: FABP4 (lipid metabolism) Control-predominant: non-AMs: Cluster 1: FABP4, CCL2, IFI6 Cluster 3: MT1G Cluster 4: SFTPC Cluster 5: VSIG4 Cluster 7: SPP1, S100A8/9 Cluster 9: BAG3 Cluster 13: SERPINB2 AMs: Cluster 12: FABP4 Cluster 14: MALAT1 Mixed: non-AMs: Cluster 6: RGS1
Baßler *et al.* 2022 48	BALF and peripheral blood from early-stage COPD	MФ3: C1QA-C, SERPINA1 (protease inhibition) MФ5 : VCAN, S100A8, CCL2, CHIT1 (monocyte attractant) MФ6 : HLA-DQ (antigen presentation) MФ7 : IFIT1, IFIT2 (viral defense) MФ8 : MKI67, TOP2A, NUSAP1, HIST1H4C, HIST1H1D (proliferation) MФ9 : HLA-DR (antigen presentation) MФ12 : HBA2, HBA1, HBB (erythrocytes or oxygen transport)
Wan *et al.* 2024 88	Lung tissues with ACOS and without ACOS	AMs: C1QA, C1QB, CD68, APOC1 IMs: LGMN, RNASE1, CCL2
Murano *et al.* 2024 102	Lung in patient with COPD and mice exposed to CS	MoAMs : LPLAT9 (pro-inflammation factor PAF)
Wohnhaas *et al.* 2024 103	Lung of eight-week-old female C57BL/6J mice with CS	MoAMs (pro-inflammation/tissue remolding) ResAMs (lipid metabolism)
Li *et al.* 2023 50	Lung tissues with non-COPD and COPD (mild/moderate)	M1 : SOD2, GPX4 (resistance to ferroptotic death) M2 : HO-1 (suspectial to ferroptotic death)

Abbreviations: AMs, Alveolar Macrophages; ACOS, Asthma-COPD Overlap Syndrome; BALF, Bronchoalveolar Lavage Fluid; COPD, Chronic Obstructive Pulmonary Disease; CS, Cigarette Smoking; IMs, Interstitial Macrophages; MoAMs, Monocyte-derived Alveolar Macrophages; ResAMs, Resident Alveolar Macrophages.
